# A Case of Brugada Syndrome Masquerading as Acute Coronary Syndrome in a Hispanic Male

**DOI:** 10.7759/cureus.35055

**Published:** 2023-02-16

**Authors:** Sudarshan Gautam, Nava R Sharma, Sajog Kansakar, Madalasa Pokhrel, Arjun Basnet

**Affiliations:** 1 Internal Medicine, Maimonides Medical Center, Brooklyn, USA; 2 Medicine, Manipal College of Medical Sciences, Pokhara, NPL; 3 Internal Medicine, Manipal College of Medical Sciences, Pokhara, NPL; 4 Internal Medicine, Montefiore Medical Center, New Rochelle, New Rochelle, USA

**Keywords:** acute st elevation myocardial infarction, brugada ekg pattern, brugada-like pattern, ventricular fibrillation, implantable cardioverter-defibrillator (icd)

## Abstract

Brugada syndrome is an aberrant ST-segment elevation in the right precordial leads. It can progress into sudden cardiac death (SCD) among patients with structurally normal hearts. Most patients are asymptomatic at presentation, but those who develop symptoms can present with syncope due to other arrhythmias such as ventricular tachycardia or fibrillation. Early diagnosis and appropriate management can prevent future complications in patients with a significant family history.

## Introduction

Brugada syndrome is a rare genetic disorder characterized by abnormal electrocardiogram (EKG) patterns and an increased risk of sudden cardiac death (SCD) [1,2]. It is caused by mutations in the sodium voltage-gated channel (5SCN5A) gene, which encodes for the sodium channel in the heart. Brugada syndrome affects approximately one in 2,000 individuals and is more common in males than in females [3,4]. Brugada syndrome diagnoses are based on specific EKG patterns, including the coved-type ST elevation in leads V1-V3 [1]. Treatment options include beta-blockers, implantable cardioverter-defibrillator (ICD) placement, and avoidance of certain medications that can worsen EKG changes [4].

This case report will describe the diagnosis and management of a 58-year-old male with Brugada syndrome after ruling out structural heart diseases.

## Case presentation

**Figure 1:**
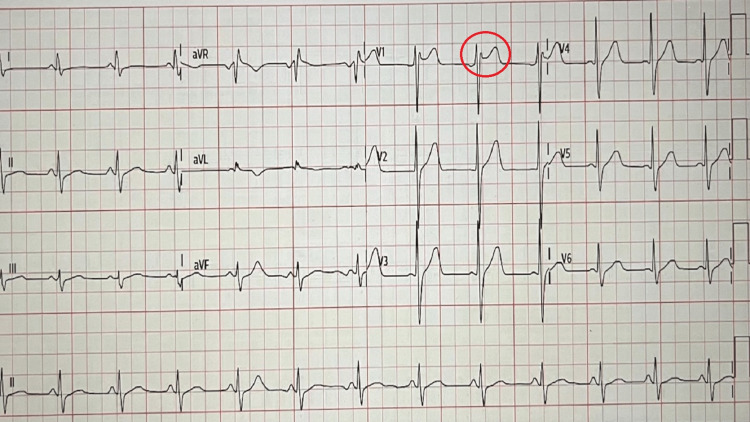
EKG on arrival: normal sinus rhythm with incomplete right bundle branch block with mild coving consistent with type II pattern. EKG: electrocardiogram

A 58-year-old Hispanic nonsmoker male patient was brought to the emergency department by paramedics after his wife found him not breathing and called 911. Paramedics found him in cardiac arrest with ventricular fibrillation. Cardiopulmonary resuscitation was performed, and spontaneous circulation was returned after two rounds of epinephrine in approximately 10 minutes. An initial EKG showed normal sinus rhythm with the right bundle branch looking like a saddleback ST elevation, as shown in Figure 1, likely to be type II Brugada.

The patient was a nonsmoker with no known past medical history. However, he had a family history of sudden death in a sister at age 12.

**Figure 2:**
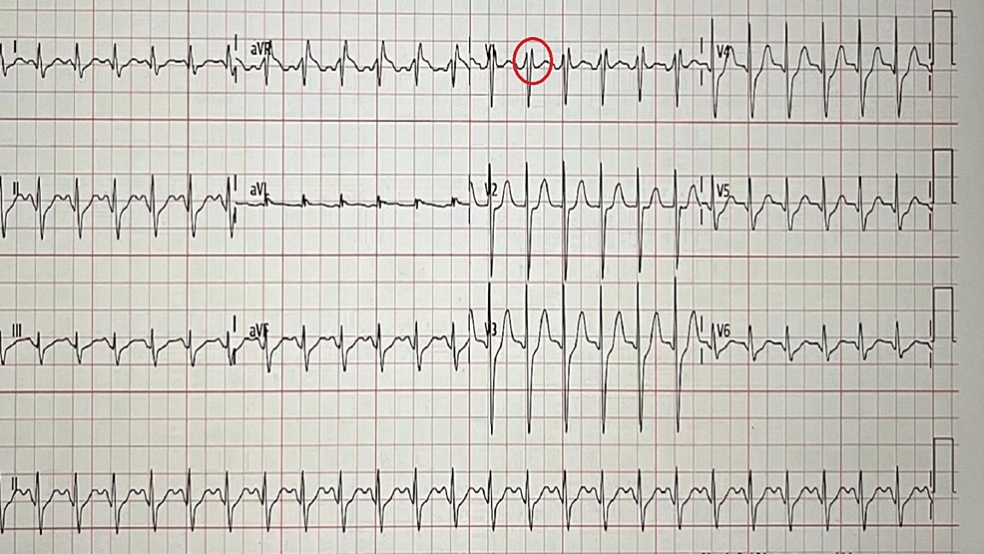
Repeat EKG: normal sinus rhythm with incomplete right bundle branch block with a type I pattern. EKG: electrocardiogram

The patient was intubated for airway protection due to altered mental status and low Glasgow Coma Scale (GCS) score. A repeat EKG six hours later was consistent with type I Brugada with ST elevation in V1 and V2, as shown in Figure 2. Troponin levels increased from an initial 0.11 to a peak of 8.32 ng/mL (normal value: 0.02-0.05 ng/mL) and finally trended back to baseline. The patient was admitted to the cardiac intensive care unit (CICU) for post-cardiac arrest care. Computed tomography (CT) scans of the head showed questionable loss of grey-white differentiation and bilateral small subarachnoid hemorrhage (SAH) in the left frontoparietal and right frontal cortex. Still, the magnetic resonance imaging (MRI) of the brain showed decreased cortical grey-white differentiation, likely reflecting the sequela of generalized hypoxic-ischemic injury. An echocardiogram showed an ejection fraction of 66%-70% with mild (grade 1) left ventricular diastolic dysfunction. The absence of clinical findings and lack of significant ST changes and echo changes helped us rule out myocardial infarction.

The patient underwent ICD placement for secondary prevention. The patient underwent a bedside percutaneous tracheostomy and was evaluated for speech and swallowing. He was transitioned to pureed foods and upgraded to easy-to-chew thin liquids. He was followed up in the cardiology outpatient clinic.

## Discussion

Brugada syndrome is a rare genetic condition that can cause sudden cardiac death in young and otherwise healthy individuals [3]. It is characterized by abnormal electrical activity in the heart, leading to a high risk of ventricular fibrillation and sudden cardiac arrest. Brugada syndrome diagnosis can be challenging, as it often requires a combination of clinical, electrocardiographic, and genetic evaluations [2]. The differential diagnosis for ST-T changes in right precordial leads includes anteroseptal ischemia, right bundle branch block, early repolarization syndrome, arrhythmogenic right ventricular cardiomyopathy, various substance abuse, and electrolyte abnormalities [5-8]. Structural heart disease must be ruled out in every case.

In this case, the patient presented with sudden cardiac arrest in ventricular fibrillation and was found to have a type II Brugada pattern on the initial electrocardiogram (EKG). However, subsequent EKG showed that the patient had a type I Brugada pattern, considered a more severe condition. The change from type II to type I pattern on EKG highlights the dynamic and evolving nature of Brugada syndrome and the importance of continuous monitoring and assessment [6].

Sudden cardiac arrest in ventricular fibrillation is a hallmark of Brugada syndrome and is a significant cause of sudden cardiac death in affected individuals [9]. Brugada syndrome diagnosis is crucial to implementing appropriate management and preventing sudden cardiac death. The management options for Brugada syndrome include implantable cardioverter-defibrillators (ICDs), anti-arrhythmic drugs, and ablation procedures [4,8]. ICDs effectively prevent sudden cardiac death in individuals with Brugada syndrome, making them the cornerstone of management.

This case report highlights the clinical presentation and management of Brugada syndrome. It emphasizes the need for a comprehensive evaluation of patients with sudden cardiac arrest in ventricular fibrillation [1,9]. A type I Brugada pattern on EKG, a positive family history, or other risk factors should increase the index of suspicion for Brugada syndrome [1,3]. Early and accurate diagnosis and appropriate management can prevent sudden cardiac death in affected individuals.

## Conclusions

Our case highlights the importance of a high index of clinical suspicion for Brugada syndrome in all patients with a family history of sudden cardiac death. ST-T changes in EKG and associated positive troponin, at times, may mimic anteroseptal ischemia due to acute coronary syndrome (ACS). A physician must be aware of this condition as early diagnosis after ruling out any precipitating causes can help guide the further management and prevention of SCD.
